# Electroacupuncture may protect pulmonary dysplasia in offspring with perinatal nicotine exposure by altering maternal gut microbiota and metabolites

**DOI:** 10.3389/fmicb.2024.1465673

**Published:** 2025-01-09

**Authors:** Yana Xie, Yang Fang, Yitian Liu, Bo Ji, Reiko Sakurai, Yifei Wang, Hewen Li, Ling Zhang, Le Wu, Tingting Guo, Ye Quan, Virender K. Rehan

**Affiliations:** ^1^School of Acupuncture-Moxibustion and Tuina, Beijing University of Chinese Medicine, Beijing, China; ^2^Lundquist Institute for Biomedical Innovation at Harbor-UCLA Medical Center, David Geffen School of Medicine at UCLA, Los Angeles, CA, United States

**Keywords:** electroacupuncture, perinatal nicotine exposure, pulmonary dysplasia, gut microbiota, short-chain fatty acids

## Abstract

**Background:**

Perinatal nicotine exposure (PNE) induces pulmonary dysplasia in offspring and it increases the risk of respiratory diseases both in offspring and across generations. The maternal gut microbiota and its metabolites, such as short-chain fatty acids (SCFAs), can regulate fetal lung development and are susceptible to nicotine exposure. Therefore, modulation of PNE-induced changes in maternal gut microbiota and SCFAs may prevent the occurrence of pulmonary dysplasia in offspring.

**Objective:**

Our previous studies demonstrated that electroacupuncture (EA) ameliorated PNE-induced impairment in offspring lung development. To further our study, we aimed to determine whether the protective effect of EA is associated with the modulation of changes in maternal gut microbiota and SCFAs.

**Methods:**

We observed changes in maternal gut microbiota and serum SCFA levels in both mother and offspring after EA treatment using a PNE rat model. Furthermore, using broad-spectrum antibiotics, we established a pseudo-germ-free PNE rat model to explore whether EA can protect offspring’s pulmonary function and lung morphology in the presence of depleted maternal gut microbiota.

**Results:**

Our study revealed that EA increased the community richness (Sobs index) of perinatal nicotine-exposed maternal gut microbiota and the abundance of beneficial bacteria (RF39, Clostridia, Oscillospirales, etc.). This was accompanied by an upregulated serum levels of acetate, butyrate, and total SCFAs in both mother and offspring rats, as well as stimulated expression of SCFA receptors (GPR41 and GPR43) in the lung tissue of offspring rats. However, the beneficial effects of EA on offspring pulmonary function (FVC, PEF, PIF, and Cdyn) and lung morphology (alveolar number and MLI) were lost after maternal gut microbiota depletion.

**Conclusion:**

These findings suggest that EA may exert its therapeutic effects on PNE-induced lung phenotype by altering maternal gut microbiota. The likely mechanism involves the associated improvement in serum SCFA levels in both mother and offspring, as well as the upregulation of SCFA receptors in the lung tissue of offspring.

## 1 Introduction

Active and passive maternal smoking during pregnancy is associated with numerous adverse pregnancy outcomes, including premature birth, intrauterine growth restriction, and neonatal respiratory system underdevelopment. These outcomes often result in pulmonary dysplasia in offspring and other lifelong respiratory consequences (McEvoy and Spindel, 2017; Ding et al., 2022; Tarasi et al., 2022). Nicotine is the principal component responsible for damaging the developing lung (Kurihara et al., 2023). Despite the well-documented harmful effects of nicotine, the global prevalence of active smoking among pregnant and lactating women is estimated to range between 5 and 40% (McEvoy and Spindel, 2017), while rates of passive smoking (secondhand smoke exposure) reach up to 75% (Zhang et al., 2015; Reece et al., 2019). Perinatal nicotine exposure (PNE) disrupts pulmonary alveolar development (Blaskovic et al., 2020) and impairs pulmonary function (Thacher et al., 2018) in offspring, leading to pulmonary dysplasia, increased susceptibility to respiratory infections (Cheemarla et al., 2019), and asthma (Ding et al., 2022). Alarmingly, these adverse effects extend to subsequent generations (Liu et al., 2020), underscoring the urgent need to address this issue.

PNE affects lung development through multiple pathways, one of which may involve the maternal gut microbiome and short-chain fatty acids (SCFAs). Prenatal nicotine exposure has been shown to alter the abundance of Firmicutes, Bacteroidetes, Proteobacteria, and Actinobacteria in maternal gut microbiota, and to reduce SCFA levels in the blood and amniotic fluid of dams (Zubcevic et al., 2022). Altered maternal gut microbiota can influence offspring lung development by regulating SCFA levels. For instance, depletion of the gut microbiome in pregnant rats using antibiotics led to decreased maternal SCFA levels, impaired placental development, and pulmonary hypoplasia in offspring (Parsons et al., 2022; Pronovost et al., 2023). Additionally, SCFAs can bind to G protein-coupled receptors (GPRs) in the lungs, thereby influencing lung development in offspring. Acetic acid supplementation was found to upregulate GPR43 gene expression in the lungs of mice with pulmonary dysplasia, reduce inflammatory factors, and mitigate lung damage (Zhang et al., 2020). Based on this evidence, we hypothesize that PNE-induced pulmonary dysplasia in offspring is at least partially due to alterations in maternal gut microbiota, SCFA levels, and GPRs expression in offspring lung.

Glucocorticoids are commonly used to prevent neonatal lung damage (Crowther et al., 2015); however, this approach has not been proven to be universally effective and may be associated with significant long-term neurodevelopmental and respiratory adverse consequences (Wang et al., 2022). Electroacupuncture (EA), a traditional Chinese medicine therapy, has been shown to regulate general well-being, immunity, and endocrine and metabolic functions, as well as to influence intestinal flora (Ye et al., 2024). Our previous studies found that EA at “Zusanli (ST36)” acupoints in PNE dams could protect offspring from pathological abnormalities in pulmonary function and lung morphology. Furthermore, we found that EA at ST36 mitigates PNE-induced pulmonary dysplasia in offspring by modulating the hypothalamic–pituitary–adrenal axis in both mother and offspring, reducing maternal glucocorticoid overexposure, and decreasing the expression of glucocorticoid receptors in the offspring’s lung (Ji et al., 2016; Liu et al., 2018; Dai et al., 2020; Lu et al., 2020). However, EA is known to improve diseases such as functional constipation (Xu H. et al., 2023; Xu M. M. et al., 2023), chronic colitis (Wang et al., 2020), myocardial ischemia reperfusion injury (Bai et al., 2021), and type 2 diabetes (An et al., 2022) by regulating the gut microbiota. Currently, it remains unclear whether EA at ST36 in perinatal nicotine-exposed dams protects offspring pulmonary dysplasia by modulating maternal gut microbiota and SCFA levels. This study aims to determine whether EA exerts its protective effect by regulating the gut microbiota of PNE dams and SCFA levels in both dams and their offspring. Additionally, we depleted the gut microbiota in PNE dams using antibiotics to observe whether the protective effect of EA on pulmonary function and lung morphology in offspring is diminished.

## 2 Materials and methods

### 2.1 Animals and breeding

Thirty-two specific pathogen free (SPF) Sprague Dawley rats (non-pregnant, 24 females with 10 weeks and 8 males with 11 weeks) from the Beijing Charles River Laboratory Animal Technology Co., Ltd. and were housed in an SPF-class animal room at the Experimental Animal Center of Beijing University of Chinese Medicine. All animals ate and drank freely, maintained on a 12:12 h light/dark cycle at a room temperature of (22 ± 2) °C, and acclimatized for 1 week. The use of experimental animals was approved by the Institutional Animal Ethics Committee of Beijing University of Chinese Medicine (BUCM-2023042901-2064) and adhered to the 3R principles (reduction, replacement, and refinement).

### 2.2 Experiment protocol

**Figure 1:**
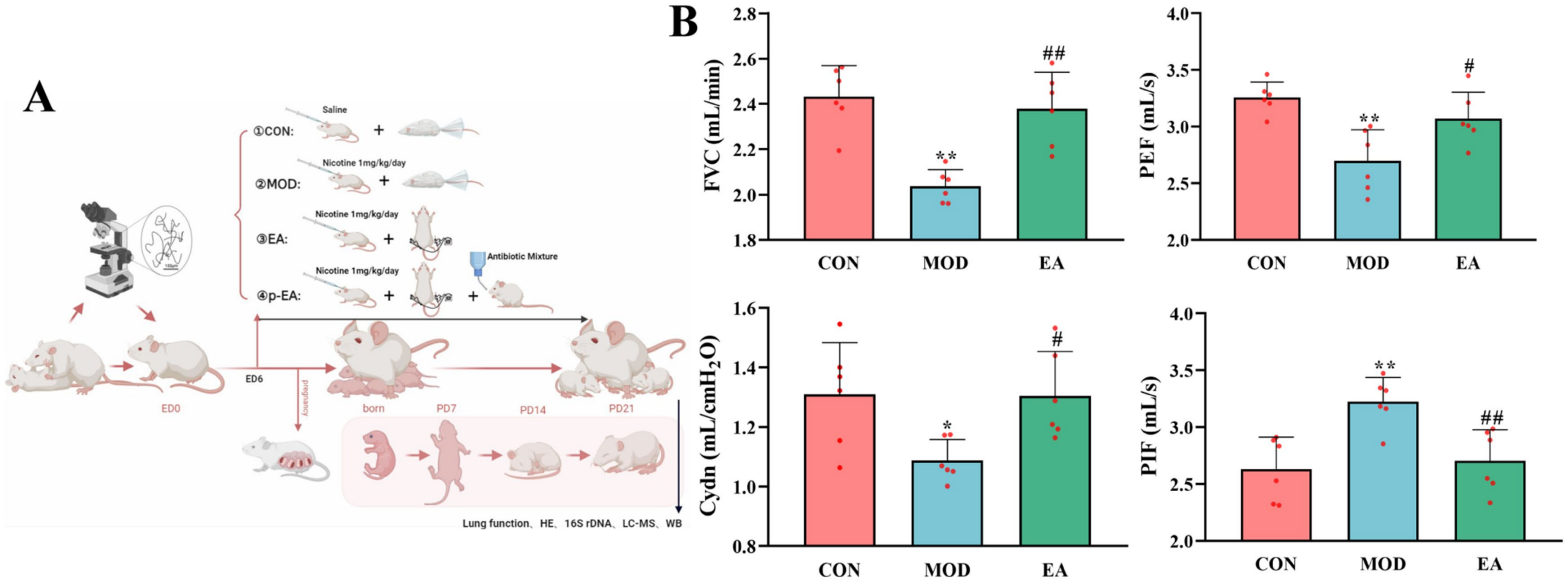
Comparison of pulmonary function in offspring rats of each group. **(A)** Experimental protocol **(B)** FVC, PEF, Cydn, and PIF, $\bar{x}$ ± s, with *n* = 6 in each group. ^*^p < 0.05, ^**^p < 0.01 vs. CON; ^#^*p* < 0.05, ^##^*p* < 0.01 vs. MOD. CON, control group; MOD, model group; EA, electroacupuncture group.

Sets of one male and three female rats were mated nightly at 18:00, and vaginal smears were collected the following morning at 7:30 to detect the presence of sperm, with the presence of sperm designated as Embryonic Day 0 (ED 0). Pregnant rats were randomly divided to one of four groups (*n* = 6 per group): (1) a saline control group (CON), (2) a nicotine model group (MOD), (3) a group treated with EA during pregnancy and lactation (EA), (4) and pseudo-germ-free + EA group (p-EA). The preparation of PNE model has been detailed in our previous studies (Ji et al., 2016; Liu et al., 2018; Dai et al., 2020; Lu et al., 2020; Ge et al., 2023). Briefly, nicotine (1 mg/kg/day, 100 μL, Sigma-Aldrich, United States) was subcutaneously injected into the neck of dams daily from ED6 to postpartum day 21 (PD21). The pseudo-germ-free experimental method followed the protocol described previously (Xu H. et al., 2023; Xu M. M. et al., 2023). Specifically, the p-EA group received a cocktail of broad-spectrum antibiotics (metronidazole, 1 mg/mL; ampicillin, 1 mg/mL; neomycin, 1 mg/mL; and vancomycin, 0.5 mg/mL; Shanghai Macklin Bio-chemical Co., Ltd., China) in sterile drinking water from ED6 to PD21. The CON and MOD groups underwent the same handling procedures, including grasping and restraining, as the EA group, but without receiving actual EA treatment (Figure 1A).

### 2.3 Electroacupuncture treatment

According to *Experimental Acupuncture Science*, ST36 is located approximately 5 mm beneath the capitulum fibulae and posterolateral to the knee joint. From ED6 to PD21 (excluding the day of delivery), the “Zusanli” acupoint on both sides of the dams were pierced to a depth of approximately 2 mm using sterile acupuncture needles (Huatuo brand). These needles were connected to the negative electrode of a HAN’s EA apparatus (LH202H, Beijing Huawei Industrial Development Company, China). Additional needles were shallowly inserted subcutaneously (2 mm beneath the ST36 acupoint on the same side) and connected to the positive electrode of the HAN’s device. Electroacupuncture was administered once daily for 20 min, with a frequency of 2/15 Hz and an intensity of 1 mA.

### 2.4 Sample size determination

An *a priori* sample size calculation, based on lung function results from a previous study (Ge et al., 2023), was conducted with an alpha level of 0.05, a power of 0.90, and an effect size of 0.9221, resulting in a sample size of six per group. Accordingly, we used six rat dams per group and collected their feces and serum. Three or four offspring rats weighing close to the median body weight were removed from each dam to measure pulmonary function, lung morphology, serum metabolites, and lung tissue protein levels.

### 2.5 Pulmonary function assessment

On PD21, the offspring rats were anesthetized with an intraperitoneal injection of 15 mg/kg Zoletil and 5 mg/kg Xylazine (Sigma-Aldrich, United States), and ventilated through tracheal intubation. The FM Animal Force Lung Function Testing System (UK EMMS) was used to evaluate the forced vital capacity (FVC), peak expiratory flow (PEF), peak inspiratory flow (PIF), and dynamic compliance of the lung (Cdyn).

### 2.6 Lung morphology assessment

At sacrifice, the left lung tissues of the offspring were removed and fixed in 4% paraformaldehyde (Coolaber). After gradient alcohol dehydration, xylene clearing, conventional paraffin embedding, and 5 μm sectioning, the sections were dewaxed, rehydrated, and stained with hematoxylin–eosin (Coolaber). Alterations in the morphology of lung tissue slices from each group were detected using a light microscope. An investigator unaware of experimental groups performed lung morphology measurements on all slices. The average number of alveoli and mean linear intercept of alveoli (MLI) were measured using Image-Pro Plus 6.0 image analysis software (Ji et al., 2016; Lu et al., 2020).

### 2.7 Fecal microbiota analysis

Fecal samples were obtained from the dams on PD21 for 16S rDNA sequencing and analysis. The FastPure Feces DNA Isolation Kit (Shanghai Major Yuhua, China) was utilized to extract bacterial DNA from the fecal microbial samples. The extracted DNA was then assessed for purity and its concentration. The V3-V4 region of the 16S rDNA was amplified using primers 338F (5′-ACTCCTACGGGAGGCAGCAG-3′)/806R (5′-GGACTACHVGGGTWTCTAAT-3′). After PCR amplification, quantification, and purification, equal amounts of the purified amplification products were mixed and ligated with sequencing adapters. Sequencing libraries were constructed using the NEXTFLEX Rapid DNA-Seq Kit (Bioo Scientific, Austin, Texas, United States), and sequencing was carried out using the Illumina PE300/PE250 platform. The resulting DNA sequences were assembled and clustered into operational taxonomic units (OTUs) at 97% similarity. Chloroplast sequences were filtered out, and the data were rarefied to minimize sequencing depth effects. Taxonomy and metagenomic function prediction were conducted using RDP Classifier (version 2.2) and PICRUSt2, respectively. Rarefaction curves and alpha diversity indices were calculated with Mothur v1.30.1, and microbial community similarity was determined by Principal Coordinates Analysis (PCoA) using the Vegan package v2.5–3 based on Bray–Curtis dissimilarity. The Linear discriminant analysis effect size (LEfSe) analysis was performed to identify significant differences in bacterial taxa abundance between groups, with an Linear discriminant analysis (LDA) score > 2 and a *p*-value < 0.05.

### 2.8 SCFAs examination

A standard mixture of SCFAs and an internal standard solution were prepared. Serum from both mother and offspring rats was processed for organic acid extraction using acetonitrile (A998-4, Fisher), and the internal standard solution was added and mixed. SCFA levels were quantified using LC-ESI-MS/MS (UHPLC-Qtrap) with the following chromatographic conditions: ExionLC AD system, Waters BEH C18 column (150*2.1 mm, 1.7 μm), column temperature at 40°C, and an injection volume of 2 μL. The mobile phases were as follows: Mobile Phase A (0.1% formic acid-water solution) and Mobile Phase B (0.1% formic acid-acetonitrile). The mass spectrometry conditions were as follows: AB SCIEX QTRAP 6500+, negative mode detection, Curtain Gas set at 35, Collision Gas set as Medium, Ion Spray Voltage at −4,500, Temperature at 450°C, Ion Source Gas1 at 40, and Ion Source Gas2 at 40. The concentration of SCFAs in the samples was calculated by substituting the peak area of the analyte in the sample into the linear equation from the calibration curve.

### 2.9 Western blotting

Proteins were extracted from lung tissues, and their concentration was determined using the BCA method (Thermo Fisher Scientific, Waltham, United States) to prepare samples. The proteins were separated by a 7.5% separating gel through electrophoresis. Following electrophoresis, the proteins were transferred onto a PVDF membrane and blocked with 5% skim milk. The membranes were incubated overnight with primary anti-bodies, including FFAR2 (1:1,000, Proteintech, Chicago, IL, United States), GPR41 (1:1,000, Thermo Fisher Scientific, Waltham, United States), and GAPDH (1:10,000, Proteintech, Chicago, IL, United States). The membranes were rinsed with TBST and treated with secondary antibody (1:10,000, Abcam, Cambridge, UK) at room temperature for 1 h. The membranes were then photographed using a gel imaging device, and the results were analyzed with Image J software.

### 2.10 Data statistics

The information on gut microbiota was analyzed and plotted using R software (version 3.3.1). The pulmonary function and morphology parameters, SCFAs and receptors data were analyzed using SPSS 20.0 (IBM, US), and visualized using GraphPad Prism 8.4.0 (GraphPad Software, Inc., CA, United States). One-way analysis of variance (ANOVA) with Bonferroni correction was employed for multiple group comparisons. Welch’s test was used for nonhomoscedastic data. The Kruskal-Wallis test was used for non-normally distributed data. Spearman correlation analyses between the relative abundance of gut microbiota and SCFAs data were conducted using NetworkX (version 1.11). Data are presented as mean ± standard deviation. *p* value of < 0.05 is statistically significant.

## 3 Results

### 3.1 Effects of EA on pulmonary function in offspring with PNE

Pulmonary function tests are key indicators of lung development in offspring. Among these, FVC reflects respiratory resistance, Cdyn reflects the elasticity of lung tissue, and PIF and PEF provide insights into respiratory muscle and pulmonary ventilation. Offspring in the MOD group showed a significant decrease in FVC (*p* < 0.01), PEF (*p* < 0.01), and Cydn (*p* < 0.05), but a significant increase in PIF compared to the CON group (*p* < 0.01). However, in the EA group, FVC (*p* < 0.01), PEF (*p* < 0.05) and Cdyn (*p* < 0.05) were significantly higher and PIF (*p* < 0.01) was significantly reduced compared with the MOD group, indicating improved lung function (Figure 1B).

### 3.2 Effects of EA on the gut microbiota in the dams with PNE

**Figure 2:**
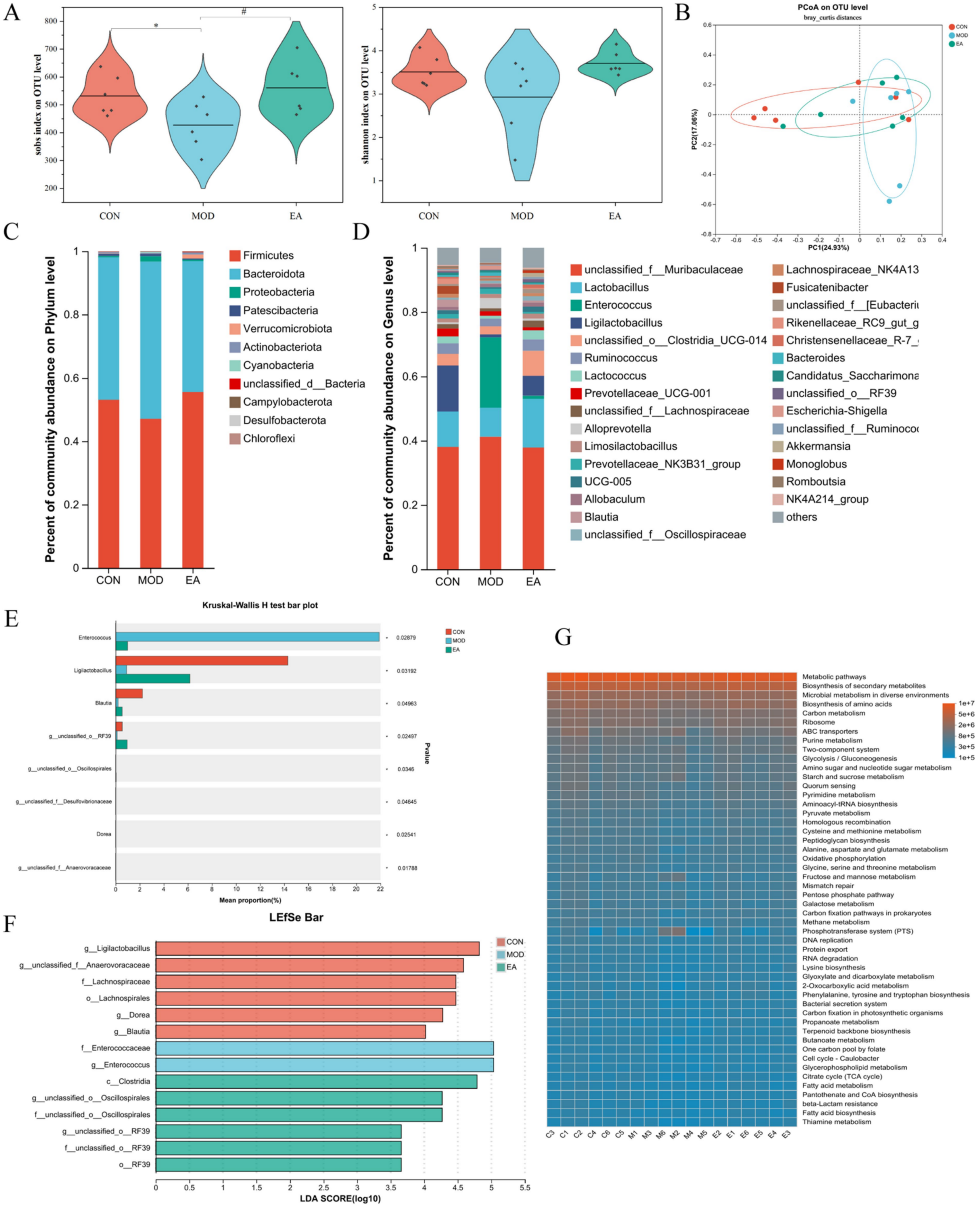
Comparison of the diversity of gut microbiota in dams of each group. **(A)** Alpha diversity analysis: Sobs and Shannon indices. Sobs symbolize community richness, and Shannon reflects community diversity. One-way ANOVA was used for statistical analysis. ^*^p < 0.05 vs. CON; ^#^*p* < 0.05 vs. MOD. CON, control group; MOD, model group; EA, electroacupuncture group. **(B)** Beta diversity analysis: PCoA based on the Bray-Curtis distance algorithm. **(C)** Phylum-level community bar plot. **(D)** Genus-level community bar plot. **(E)** Significant difference analysis at the genus level. Kruskal-Wallis H test, ^*^p < 0.05. **(F)** LDA score distribution histogram for LEfSe on taxonomic levels ranging from phylum to genus. Multiple group comparison strategy: all-against-all. LDA score > 2, *p* < 0.05. The larger the LDA scores, the stronger the influence of species abundance on the difference. **(G)** Functional prediction using PICRUSt2. *n* = 6 in each group.

The sequencing output data for the samples are shown in Supplementary Table S1. The rank-abundance curve showed that the distribution of gut microbial species was relatively homogeneous across all three groups. However, the gut microbiota in the CON and EA groups exhibited higher levels of abundance compared to the MOD group (Supplementary Figure S2A). Alpha diversity primarily reflects the richness and diversity of microbial communities. Compared with the CON group, the Sobs index was significantly decreased in the MOD group (*p* < 0.05), while the Shannon index decreased but not significantly (*p* > 0.05). The EA group showed a significant increase in the Sobs index compared to the MOD group (*p* < 0.05), and although the Shannon index also increased, this change was not significant (*p* > 0.05) (Figure 2A).

Beta diversity measures the similarity or dissimilarity of community structures among different groups. The PCoA plot showed that the gut microbiota in the three groups was not entirely distinct, but that of the EA group was more similar to the CON group compared to the MOD group (Figure 2B). Partial least squares-discriminant analysis (PLS-DA) confirmed that the gut microbiota differed among the three groups (Supplementary Figure S2B). The Venn diagram illustrated the shared and unique OTUs in each group. The CON and MOD groups shared 89 OTUs, the CON and EA groups shared 274 OTUs, and the MOD and EA groups shared 146 OTUs (Supplementary Figure S2C).

The community composition at the phylum level revealed that the dominant bacteria in the gut microbiota were Firmicutes, Bacteroidota, Proteobacteria, and Actinobacteriota, which together accounted for over 98% of the community. In the CON group, microbiota was enriched in Firmicutes (53.09%), Bacteroidota (44.96%), Proteobacteria (0.45%) and Actinobacteriota (0.38%). In comparison, the MOD group showed a decrease in the abundance of Firmicutes (47.09%) and Actinobacteriota (0.34%), while the abundance of Bacteroidota (49.65%) and Proteobacteria (1.67%) increased, though these differences were not statistical significant (*p* > 0.05). In the EA group, there was an increase in Firmicutes (55.53%) and Actinobacteriota (0.60%), with a decrease in Bacteroidota (41.38%) and Proteobacteria (0.42%) compared to the MOD group, though these changes were not statistically significant (*p* > 0.05) (Figure 2C; Supplementary Figure S2D).

At the genus level, we present the composition and relative abundance of the top 30 genera in the three groups (Figure 2D). Species difference analyzes showed variations in abundance of *Enterococcus*, *Ligilactobacillus*, *Blautia*, *g__unclassified_o__RF39*, *g__unclassified_o__Oscillospirales*, *g__unclassified_f__Desulfovibrionaceae*, *Dorea*, and *g__unclassified_f__Anaerovoracaceae* across the three groups. Specifically, *Ligilactobacillus*, *Blautia*, *g__unclassified_o__RF39*, *g__unclassified_o__Oscillospirales*, *Dorea*, and *g__unclassified_f__Anaerovoracaceae* had elevated relative proportions in the CON and EA groups, while *g__unclassified_f__Desulfovibrionaceae* had a higher relative abundance in the CON group, and *Enterococcus* showed an elevated relative proportion in the MOD group (Figure 2E).

LEfSe analysis was conducted at multiple taxonomic levels, ranging from phylum to genus (Supplementary Figure S2E). The LDA results showed that certain taxa were enriched in distinct groups. The CON group had greater amounts of *g__Ligilactobacillus*, *g__unclassified_f__Anaerovoracaceae*, f__Lachnospiraceae, o__Lachnospirales, *g__Dorea*, and *g__Blautia*. The MOD group exhibited substantially higher levels of f__Enterococcaceae and *g__Enterococcus*, while c__Clostridia, *g__unclassified_o__Oscillospirales*, f__unclassified_o__Oscillospirales, *g__unclassified_o__RF39*, f__unclassified_o__RF39, and o__RF39 were primarily enriched in the EA group (Figure 2F). PICRUSt2 was utilized to analyze the KEGG metabolic functions across various samples. The results indicated that several SCFA productions and metabolic pathways, such as Glycolysis/Gluconeogenesis, Starch and Sucrose metabolism, Pyruvate metabolism, Pentose phosphate pathway, Citrate cycle, Propanoate metabolism, Butanoate metabolism, were dominant (Xu et al., 2020) (Figure 2G).

### 3.3 Effects of EA on serum SCFA levels in dams with PNE

**Figure 3:**
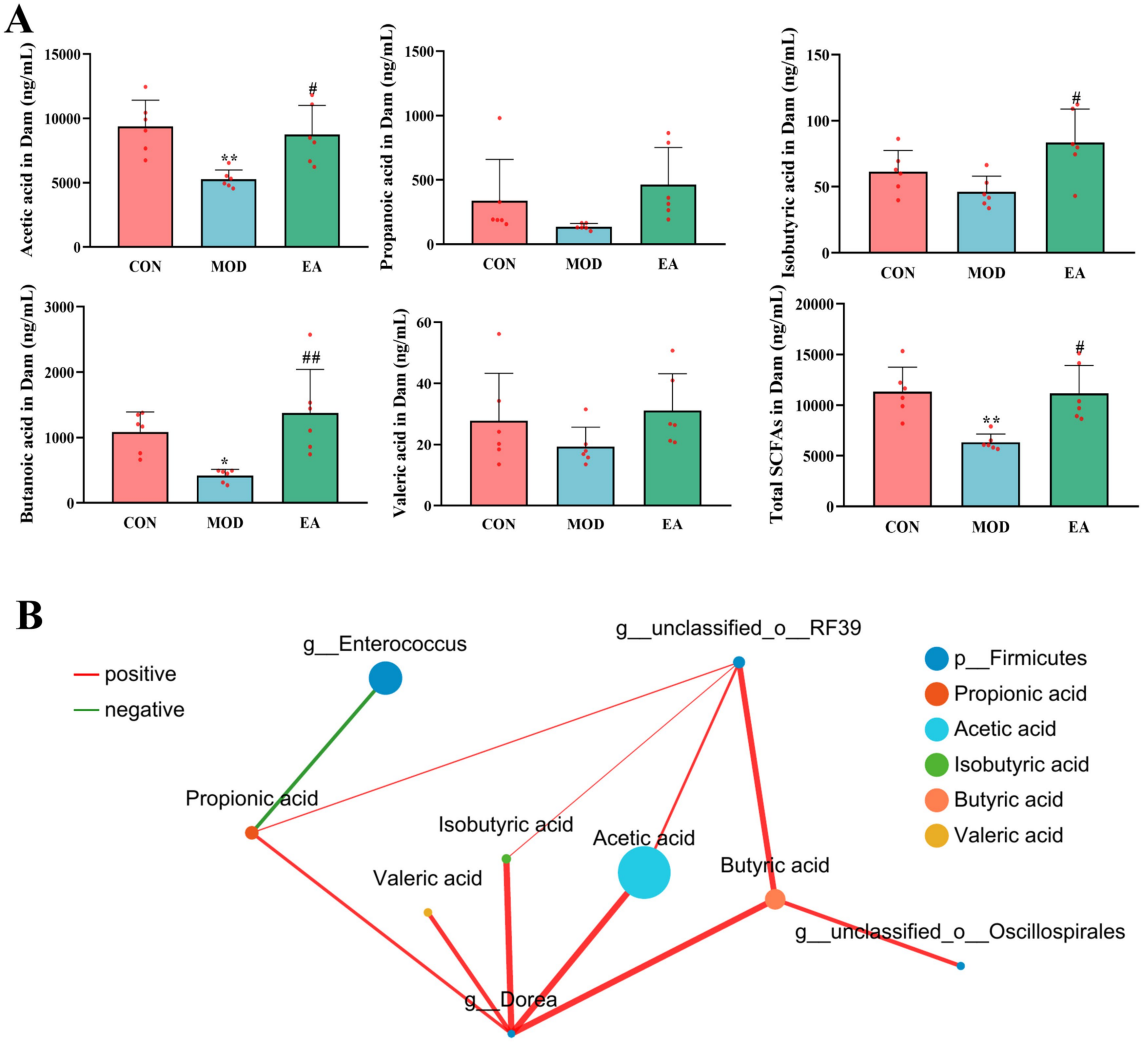
Comparison of serum SCFA levels in dams of each group. **(A)** Serum short-chain fatty acid levels in dams of each group, $\bar{x}$ ± s, *n* = 6 in each group. ^**^p < 0.01 vs. CON; ^#^*p* < 0.05, ^##^*p* < 0.01 vs. MOD. CON, control group; MOD, model group; EA, electroacupuncture group. **(B)** The co-occurrence networks were constructed to explore the relationships between serum SCFAs and the differential microbiota at the genus level of dams’ feces. A statistically robust correlation between two nodes is indicated by a Spearman’s correlation coefficient greater than 0.5 or less than −0.5, and a *p*-value below 0.05. Node size represents species abundance, different colors represent different species or metabolites. Red lines indicate positive correlations, while green lines represent negative correlations. The thicker the line, the higher the correlation, and the more connections between nodes indicate closer relationships.

Compared with the CON group, serum levels of acetic acid (*p* < 0.01), butyric acid (*p* < 0.05), and total SCFAs (*p* < 0.01) were significantly reduced in the dams of the MOD group, while propionic acid, isobutyric acid, and valeric acid were non-significantly decreased (*p* > 0.05). In contrast, the dams of the EA group showed significantly higher serum levels of acetic acid (*p* < 0.05), isobutyric acid (*p* < 0.05), butyric acid (*p* < 0.01), and total SCFAs (*p* < 0.05) compared to the MOD group, while levels of propionic acid and valeric acid remained unchanged (*p* > 0.05) (Figure 3A).

Further analysis of the relationship between gut microbiota and SCFAs revealed that at the genus level, *Dorea* was positively correlated with five SCFAs, *g__unclassified_o__Oscillospirales* was positively correlated with butyric acid, *g__unclassified_o__RF39* was positively correlated with acetic acid, butyric acid, isobutyric acid, and propionic acid. In contrast, *g__Enterococcus* showed a negative correlation with propionic acid (Figure 3B).

### 3.4 Effects of EA on serum SCFA levels and SCFA receptors in offspring with PNE

**Figure 4:**
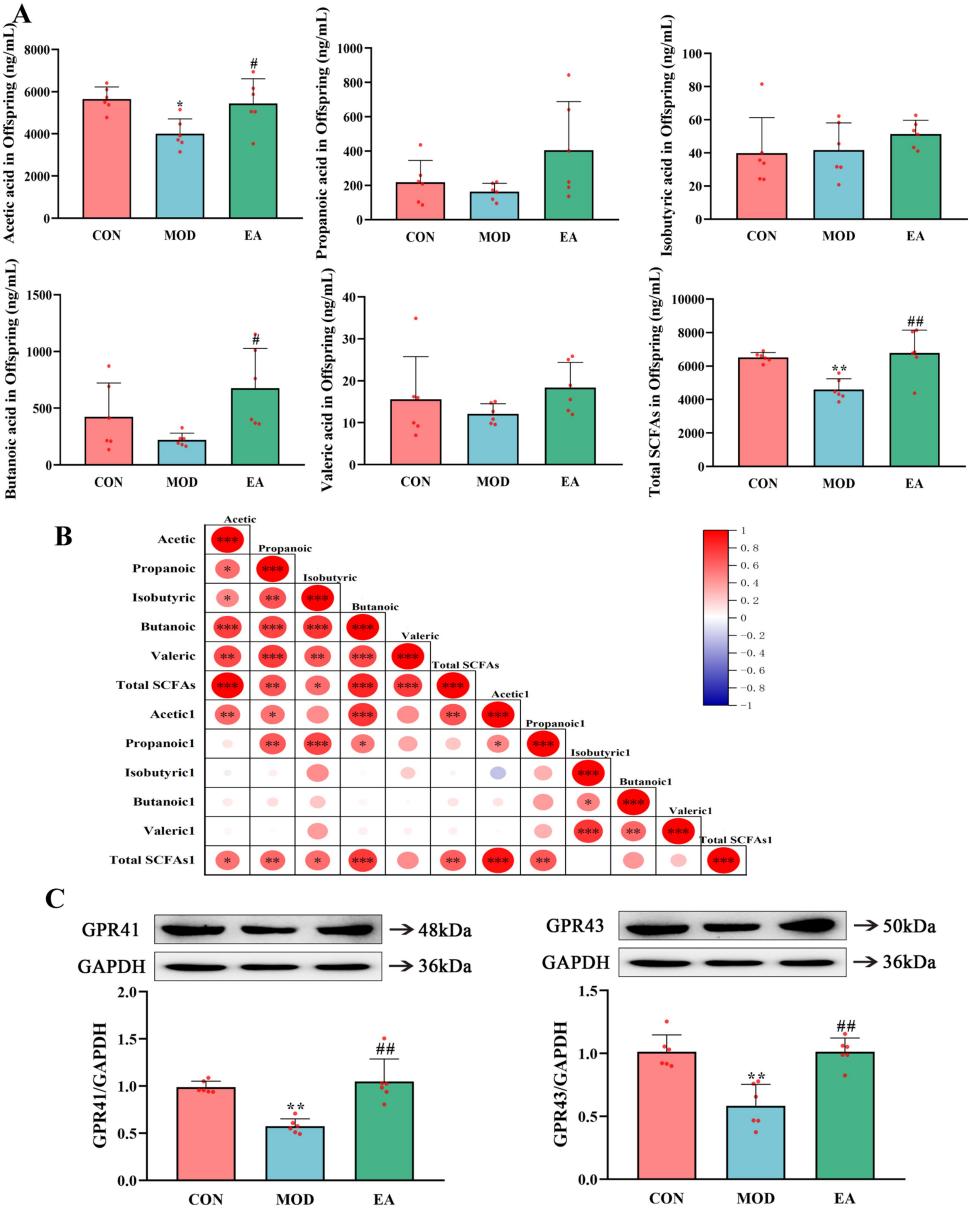
Comparison of serum SCFA levels and SCFA receptors in offspring rats of each group. **(A)** Serum short-chain fatty acid levels in offspring rats of each group. **(B)** Matrix plot of Pearson correlation analysis of serum SCFA levels between mother rats and their offspring. Acetic_acid represents maternal serum acetic acid content, while Acetic_acid1 represents offspring serum acetic acid content, and so on. Red: positive association; blue: negative association. The color intensity and size of the circle show the degree of association, ^*^p < 0.05, ^**^p < 0.01, ^***^p < 0.001. **(C)** The expression of SCFA receptors GPR41 and GPR43 in PNE offspring lung tissues, $\bar{x}$ ± s, *n* = 6 in each group. ^**^p < 0.01 vs. CON; ^#^*p* < 0.05, ^##^*p* < 0.01 vs. MOD. CON, control group; MOD, model group; EA, electroacupuncture group.

Compared with the CON group, serum levels of acetic acid and total SCFAs were significantly lower (*p* < 0.05) in the offspring rats of the MOD group, accompanied by reduced expression of GPR41 and GPR43 in lung tissues (*p* < 0.01). However, there were no significant differences in levels of propionic acids, isobutyric acids, butyric acids, and valeric acids (*p* > 0.05). In contrast, offspring rats in the EA group exhibited significantly elevated serum levels of acetic acid (*p* < 0.05), butyric acid (*p* < 0.05), and total SCFAs (*p* < 0.01) compared to the MOD group. They also showed increased expression of GPR41 and GPR43 (*p* < 0.01). No significant differences were noted in the levels of propionic acid, isobutyric acid, and valeric acids (*p* > 0.05) (Figures 4A,C). Correlation analysis demonstrated a positive relationship between serum SCFA levels in the dams and offspring (Figure 4B).

### 3.5 Effects of EA after depleting the dams’ gut microbiota on PNE-induced changes in offspring pulmonary function and lung morphology

**Figure 5:**
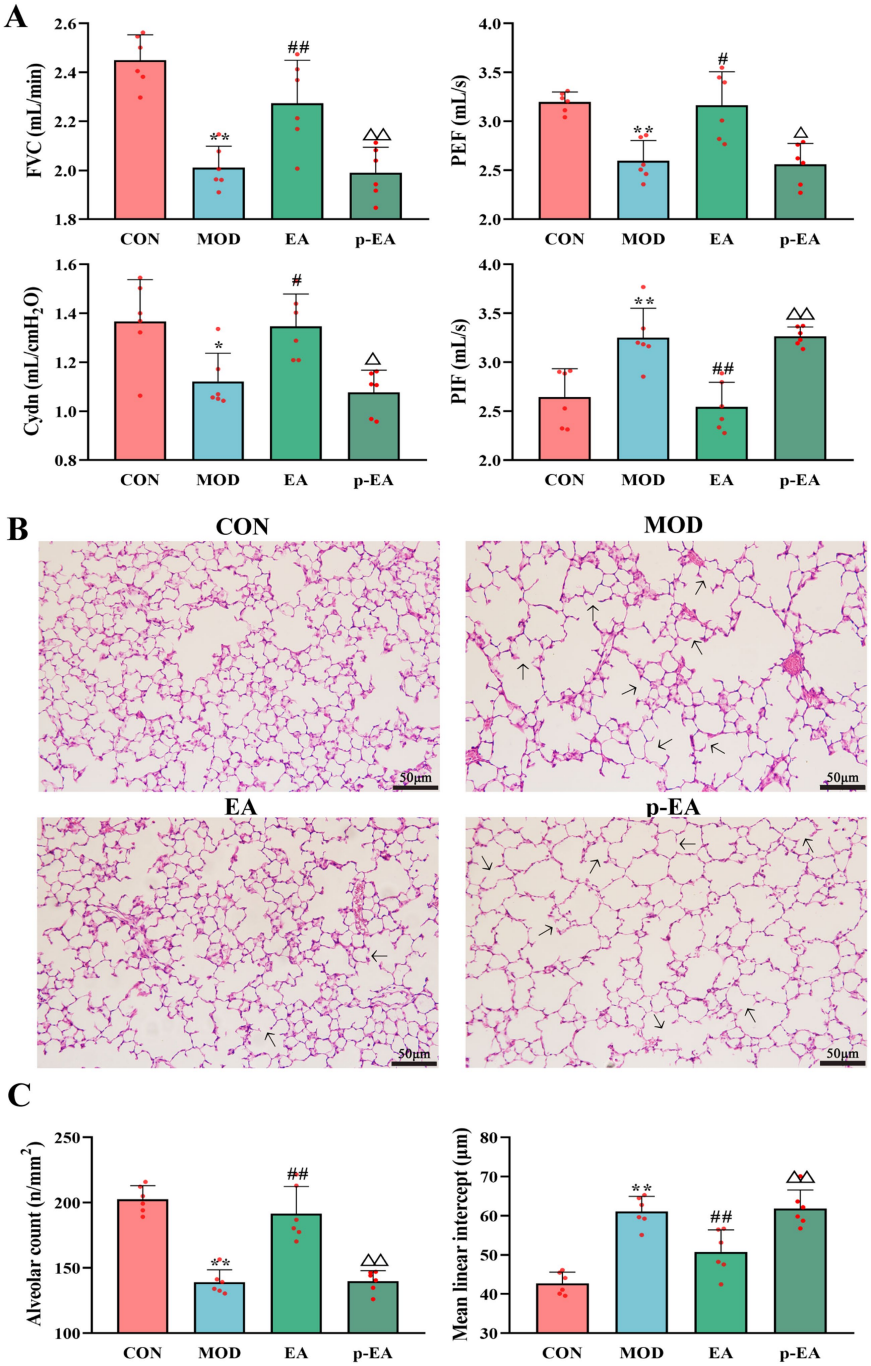
Comparison of pulmonary function and lung morphology in offspring rats of each group. **(A)** FVC, PEF, Cydn, and PIF. **(B)** HE staining of lung tissues in offspring rats of each group, ×200. **(C)** Number and MLI of alveoli, $\bar{x}$ ± s, *n* = 6 in each group. ^**^p < 0.01 vs. CON; ^#^*p* < 0.05, ^##^*p* < 0.01 vs. MOD; ^△^*p* < 0.05, ^△△^*p* < 0.01 vs. EA. CON, control group; MOD, model group; EA, electroacupuncture group; p-EA, pseudo-germ-free + EA group.

We investigated the role of dams’ gut bacteria in the effects of EA by eliminating with antibiotics. The concomitant use of antibiotics in the p-EA group blocked the protective effects of EA on pulmonary function and lung morphology in the offspring. This was evident as FVC (*p* < 0.01), PEF (*p* < 0.05) and Cydn (*p* < 0.05) were significantly reduced, while PIF (*p* < 0.01) increased. Additionally, normal lung morphology was not restored, with alveolar number and MLI resembling those in the MOD group (Figure 5).

## 4 Discussion

In addition to controlling nutrient supply for growth, a mother can influence the development of her offspring’s lungs through various mechanisms. These include regulation of the HPA axis (Liu et al., 2018), gut microbiota and its metabolites (Rodrigues et al., 2021), genetics (Shorey-Kendrick et al., 2017), and immune mechanisms (Zhao et al., 2019). Recent studies have shown that nicotine, the primary harmful component of tobacco smoke, can alter the maternal gut microbiota and the levels of SCFA in both mother and offspring. These changes may affect the developing lungs of the offspring (Zhang et al., 2020; Zubcevic et al., 2022). In our previous work, we demonstrated that applying conventional EA at ST36 acupoints in the PNE dam protects their offspring from PNE-induced pulmonary dysplasia (Liu et al., 2018; Lu et al., 2020). Notably, this protective effect was also observed when EA was applied at a shallow depth on the ST36 acupoints (unpublished data). The pulmonary function data from this study supports these findings. Consistent with recent research suggesting that EA’s therapeutic effects may be due to its modulation of gut microbiota and SCFA levels (Xu H. et al., 2023; Xu M. M. et al., 2023), our current work highlights the significant role of maternal gut microbiota and the SCFA levels in both mother and offspring in mediating EA’s protective effects against PNE-induced pulmonary dysplasia.

Considered as the largest “microbial organ” in the human body, the gut microbiota consists of beneficial bacteria, pathogenic bacteria, and conditional pathogenic bacteria. It plays a crucial role in maintaining host health and contributing to disease development (Hou et al., 2022). Numerous studies have revealed that maternal gut microbiota may be involved in placental morphogenesis, offspring growth and development, and have a significant impact on the long-term respiratory health of offspring (Lange et al., 2012; Thorburn et al., 2015; Sato et al., 2019; Lopez-Tello et al., 2022; Pronovost et al., 2023). In a study involving pregnant rats, nicotine exposure (6 mg/kg) resulted in notable changes in the relative abundance of microbial taxa in the cecum. Specifically, there was an increased relative abundance of Actinobacteria and Firmicutes, a downward trend in the relative abundance of Bacteroides and Proteobacteria, and a significant enrichment of the genus *SMB53* within the Firmicutes species observed on day 19 of gestation (Zubcevic et al., 2022). These findings suggest that nicotine exposure during pregnancy leads to alterations in the maternal gut microbiota, which we hypothesize may contribute to pulmonary dysplasia in the offspring.

In this study, we employed a well-established PNE dam model to induce offspring pulmonary dysplasia. Maternal fecal microbiota was analyzed on PD21 to evaluate the impact of PNE on gut microbiota. Our findings revealed that PNE altered gut microbiota, as evidenced by a reduction in the community gut microbiota richness, reflected by the Sobs index. In addition, there was a downward trend in the relative abundance of Firmicutes and Actinobacteria, while the relative abundance of Bacteroidetes and Proteobacteria increased slightly, though not significantly. At the genus level, we observed a significant enrichment of *Enterococcus* in the family Enterococcaceae of Firmicutes, alongside a reduction in the abundance of *Dorea*, *Ligilactobacillus*, and *Blautia* compared to the CON group. Notably, *Enterococcus* is a conditionally pathogenic bacterium found in higher concentrations in the gut microbiota of smokers (Northrup et al., 2021). Its enrichment has been associated with various pathological conditions, including an increased predisposition to infections and wheezing (Lange et al., 2012; Fiore et al., 2019). Conversely, we observed a decrease in the abundance of *Dorea*, a genus linked to the inhibition of nicotine addiction and its reduced prevalence in children with allergic rhinitis (Chen et al., 2023; Chiu et al., 2019). *Ligilactobacillus* and *Blautia* are known to produce beneficial metabolites such as acetic acid and lactic acid, making them popular candidates for probiotics (Zheng et al., 2020; Liu et al., 2021). Overall, our data demonstrate that PNE alters the maternal gut microbiota, reducing its richness, and significantly enriching the conditionally pathogenic bacteria, while decreasing the abundance of beneficial bacterial genera. Notably, the changes we observed in the fecal microbiota of PNE dams differ somewhat from those reported in a previous study (Zubcevic et al., 2022). These differences may be attributable to differences in nicotine dosage (1 mg/kg vs. 6 mg/kg), delivery method (subcutaneous injection vs. osmotic minipump), exposure duration (E6 to PD21 vs. E6 to E21), and timing of microbiota determination (PD21 vs. E19). Additionally, there were differences in the testing methods employed, such as the use of different primer sets (338F-806R vs. 515F-806R) for sequence identification.

Shallow EA needling at ST36 in PNE dams partially restored gut microbiota structure in the dams. This restoration was evidenced by an increase in the Sobs index and a reversal in phylum-level variations induced by PNE, indicating a partial reduction of PNE-induced alterations in maternal gut microbiota. Moreover, the resultant phylum-level changes resulting from EA were similar to those in the gut microbiota of individuals who had quit smoking (Biedermann et al., 2014). Additionally, EA promoted the enrichment of o__RF39, Clostridia, and Oscillospirales within the Firmicutes phylum of the maternal gut microbiota affected by PNE. At the genus level, EA decreased the abundance of *Enterococcus* while increasing the abundance of *Ligilactobacillus*, *Blautia*, and *Dorea*. The order RF39, a recently defined order of Bacilli, has limited relevant reports available (Kim et al., 2022), but one study demonstrated a negative correlation of Bacilli abundance and the occurrence of asthma (Li et al., 2024). *Clostridium*, a member of the Clostridia class, and *Oscillospira*, belonging to the Oscillospirales family, are known to upregulate butyric acid levels and are considered potential candidates for probiotics (Guo et al., 2020; Yang et al., 2021). In a broiler lung injury model induced by PM2.5 exposure, a decrease in the abundance of Oscillospirales in the gut microbiota was observed, along with a reduction in SCFAs production, which may be associated with lung injury (Zhou et al., 2024). Based on these findings, we conclude that shallow EA needling at ST36 in PNE dams can exert a protective effect against offspring pulmonary dysplasia. This protective effect is likely mediated through the regulation of the maternal gut microbiota composition and structure, including an increase in community richness and abundance of beneficial bacteria, and reduced prevalence of opportunistic pathogenic bacteria.

SCFAs are among the most abundant microbial metabolites in the gut, consisting primarily of acetic, propionic, and butyric acids. Adverse changes in maternal serum SCFA levels may affect developing offspring and predispose them to respiratory diseases (Priyadarshini et al., 2014; Thorburn et al., 2015). Nicotine exposure in pregnant rats has previously been shown to decrease propionic acid levels in cecum and cause a downward trend in plasma acetic, propionic and butyric acids concentrations (Zubcevic et al., 2022). In our study, we also found decreased serum levels of acetic acid, butyric acid, and total SCFAs in PNE dams, with a downward trend in propionic, isobutyric and valeric acid levels. However, shallow EA needling at ST36 in PNE dams increased serum levels of acetic acid, isobutyric acid, butyric acid, and total SCFAs, while showing an upward trend in propionic and valeric acid concentrations. These results indicate that EA may protect offspring from PNE-induced pulmonary dysplasia by improving maternal SCFA levels.

Correlation network analysis between maternal gut microbiota and serum SCFAs revealed the following associations: *Enterococcus* was negatively correlated with propionic acid; *g__unclassified_o__Oscillospirales* was positively correlated with butyric acid; *g__unclassified_o__RF39* was positively correlated with acetic, butyric, isobutyric and propionic acids; and *Dorea* was positively correlated with acetic, butyric, isobutyric, propionic and valeric acids. Since *Enterococcus* is not a major producer of SCFAs (Akhtar et al., 2022; Zhang et al., 2023) and the levels of acetic, butyric, and total SCFAs in the serum of PNE dams significantly altered without a statistically significant decrease in propionic acid levels, it suggests that PNE impairs offspring lung development likely by increasing the abundance of conditionally pathogenic bacteria and reducing the abundance of acetic or butyric acid producing bacteria. As a result, the protective effects of EA may be related to its ability to reverse these gut microbiota changes in PNE dams.

Moreover, changes in SCFA levels in early life of offspring may influence the development of later respiratory diseases. For example, a clinical trial found that infants with higher levels of butyric and propionic acid in their stool had a lower risk of future asthma (Roduit et al., 2019). SCFAs can bind to GPRs in the lung, influencing offspring lung development, with acetic acid and propionic acid preferentially activating GPR43, and propionic acid and butyric acid activating GPR41 (Mishra et al., 2020; Zhang et al., 2020). In the present study, we found that PNE led to decreased serum levels of acetic acid and total SCFAs, as well as a downward trend in the levels of propionic, isobutyric, butyric and valeric acids in the offspring of exposed dams. Correlation analysis indicated that this may be related to the reduced levels of SCFA in the serum of the dams. Additionally, PNE also downregulated GPR41 and GPR43 expression in offspring lungs. Therefore, reduced receptor binding of already diminished SCFAs provides an additional strong plausible contributor to PNE-induced offspring pulmonary dysplasia. Reversal of these effects by EA suggests that EA mediated amelioration of PNE-induced pulmonary dysplasia in offspring may involve modulation of serum SCFA levels in both the dams and offspring and the increased expression of SCFA receptors in offspring lung tissue.

Finally, since antibiotics can result in gut microbiome depletion, we used an antibiotic cocktail (Abx), consisting of ampicillin, neomycin, metronidazole and vancomycin, to further clarify the role of maternal gut microbiota in EA-mediated protection against PNE-induced offspring lung injury. Notably, a rodent study showed that, along with the reduction in gut microbiome abundance and diversity, Abx also reduced serum acetic, propionic and butyric acid levels (Ray et al., 2021). We observed that Abx administration, when combined with EA, blocked the beneficial effects of EA on pulmonary function and lung morphology in PNE offspring. These findings suggest that the protective effects of EA against PNE-induced pulmonary impairments in offspring may be related to maternal gut microbiota.

## 5 Conclusion

These data show that shallow EA needling at ST36 in PNE dams effectively improves pulmonary dysplasia in offspring rats. The protective mechanism of EA is likely associated with the regulation of changes in the gut microbiota of PNE dams. This mechanism primarily involves enhancing the composition of the maternal gut microbiota by increasing the community richness, boosting the abundance of beneficial bacteria, reducing the abundance of conditionally pathogenic bacteria, and upregulating the levels of acetic acid, butyric acid and total SCFAs in serum of dams and offspring. Additionally, EA treatment promotes the expression of GPR41 and GPR43 in offspring lung tissue. However, several limitations exist in our study. We failed to perform fecal transplantation to further elucidate the relationship between the therapeutic effect of EA and the gut microbiota and SCFAs. Moreover, we also did not use untargeted metabolomics or other methods to determine whether EA caused changes in other metabolites in the serum. Furthermore, the long-term effects of EA on the lung development of offspring were not investigated in our study, which is a long process that can be extended into until adolescence. For this reason, further studies are required needed to fully comprehend the specific mechanisms underlying the effects of EA treatment on gut microbiota and SCFAs, as well as the long-term efficacy of EA.

## Data Availability

The data presented in the study are deposited in the NCBI Sequence Read Archive (SRA) repository, accession number SRR 31430113-31430130.
