# Supplementary material for: Electroacupuncture may protect pulmonary dysplasia in offspring with perinatal nicotine exposure by altering maternal gut microbiota and metabolites
Source: Front Microbiol. 2025 Jan 9;15:1465673. doi: 10.3389/fmicb.2024.1465673 (PMC11754296; doi:10.3389/fmicb.2024.1465673)
Supplement: Supplementary file 1 [file Data_Sheet_1.docx]

Supplementary Material

# Supplementary Table

Table 1. Sequencing output information

| Sample | reads | Bases | avg | min | max | Sample | reads | bases | avg | min | max |
| --- | --- | --- | --- | --- | --- | --- | --- | --- | --- | --- | --- |
| Con_1 | 63134 | 26580754 | 421.021225 | 202 | 441 | Mod_4 | 64801 | 27351973 | 422.091835 | 261 | 524 |
| Con_2 | 62571 | 26277500 | 419.962922 | 318 | 469 | Mod_5 | 62920 | 26469416 | 420.683662 | 262 | 431 |
| Con_3 | 65077 | 27447633 | 421.77164 | 202 | 435 | Mod_6 | 54301 | 23059353 | 424.657981 | 298 | 445 |
| Con_4 | 62607 | 26254860 | 419.359816 | 216 | 432 | EA_1 | 59464 | 24988620 | 420.231064 | 231 | 434 |
| Con_5 | 68018 | 28604159 | 420.538078 | 203 | 521 | EA_2 | 63210 | 26602983 | 420.866682 | 320 | 444 |
| Con_6 | 60667 | 25577587 | 421.60626 | 203 | 436 | EA_3 | 59238 | 24916642 | 420.619231 | 224 | 431 |
| Mod_1 | 59338 | 25165365 | 424.102009 | 338 | 527 | EA_4 | 61058 | 25763907 | 421.957925 | 227 | 431 |
| Mod_2 | 45514 | 19388474 | 425.989234 | 255 | 432 | EA_5 | 67669 | 28234499 | 417.244218 | 261 | 450 |
| Mod_3 | 67986 | 28561550 | 420.109287 | 203 | 456 | EA_6 | 64766 | 27100457 | 418.436479 | 261 | 509 |

Note: Sample: the name of the sample, reads: number of sequences, Bases: number of bases, avg: average length, min: shortest sequence length, max: longest sequence length.

# Supplementary Figure


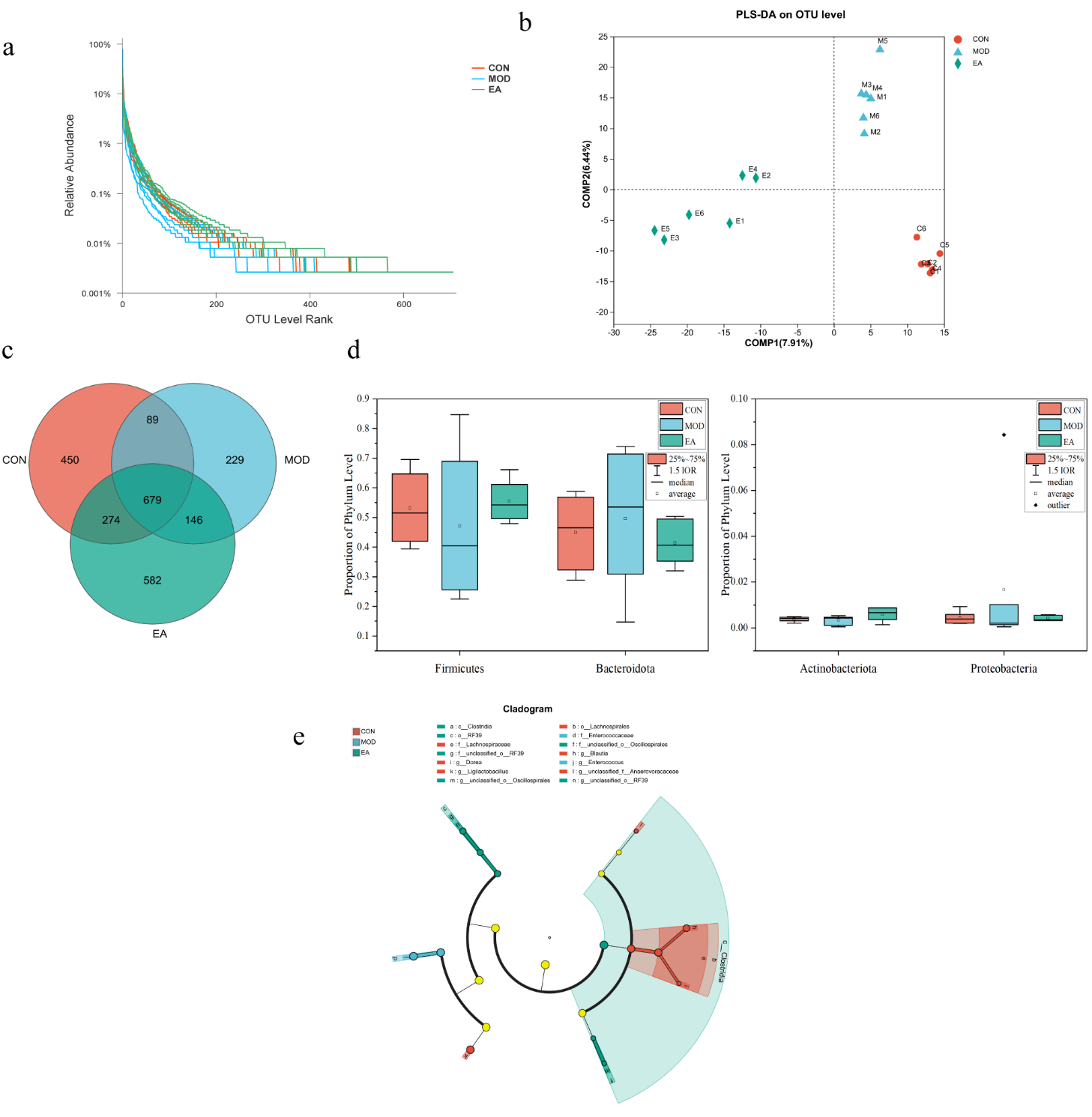


**Supplementary Figure 2.** Gut microbiota in dams of each group. (a) Rank-Abundance curve. (b) Sample grouping analysis: Partial Least Squares Discriminant Analysis (PLS-DA). (c) Annotated Venn diagram analysis. Different colors represent different groups, and the numbers in the overlapping parts represent the number of species shared by multiple groups. The numbers in the non-overlapping parts represent the number of species specific to each corresponding group. (d) Box plots of Firmicutes, Bacteroidota, Proteobacteria, and Actinobacteriota. The Kruskal-Wallis H test was used for analysis. (e) The Linear discriminant analysis effect size (LEfSe) on taxonomic levels ranging from phylum to genus. Nodes of different colors indicate significantly enriched microbial taxa within corresponding groups and exerting significant effects on inter-group differences; pale yellow nodes indicate microbial taxa with no significant differences among different groups or no significant effects on inter-group differences.
